# Novel Ablation Catheters for Atrial Fibrillation

**DOI:** 10.31083/j.rcm2505187

**Published:** 2024-05-23

**Authors:** Iwanari Kawamura, Jacob Koruth

**Affiliations:** ^1^Helmsley Electrophysiology Center, Department of Cardiology, Icahn School of Medicine at Mount Sinai, New York, NY 10029, USA; ^2^Department of Cardiovascular Medicine, Tokyo Medical and Dental University, 113-8519 Tokyo, Japan

**Keywords:** electroporation, atrial fibrillation, pulmonary vein isolation, catheter ablation

## Abstract

Various ablation technologies with different energy sources are currently being either used or being investigated for atrial fibrillation (AF) ablation. Potential complications continue to occur due to the indiscriminate thermal effects on non-targeted tissues adjacent to the myocardium that are common to all thermal ablation modalities. Pulsed field ablation (PFA) has recently gained significant attention and interest as an approach to AF ablation. PFA uniquely has the ability to circumvent certain complications related to thermal energy. PFA is a non-thermal ablation modality with the potential for unique-tissue selectivity that can minimize damage to collateral cardiac structures. Several PFA systems for AF ablation are currently being investigated. Some PFA systems have been designed to serve as single-shot approaches to achieve pulmonary vein isolation (PVI), and others have focal designs enabling flexible PVI lesion sets as well as linear/focal ablations. Favorable acute success rates and low incidence of complications with short procedure times have been reported with several PFA systems regardless of catheter design (single-shot or focal catheter). Clinical PFA studies in which chronic remapping was conducted, demonstrated pulmonary vein (PV) durability improved with evolutional modifications of pulsed field waveforms/dosing, achieving over 90% PV durability with optimized waveforms. Rare adverse events related to PFA may surface with its increasing use worldwide and as sicker patients get exposed to PFA. We believe that both excitement and vigilance are in order as we embark on yet another new chapter of AF ablation.

## 1. Introduction

Pulmonary vein isolation (PVI) with various technologies using different energy sources has demonstrated its safety and effectiveness in treating patients with atrial fibrillation (AF) [1]. However, complications continue to occur with thermal ablation modalities such as radiofrequency ablation (RFA), cryoballoon ablation, and laser ablation that include pulmonary vein (PV) stenosis, phrenic nerve palsy, and atrio-esophageal fistulas [2, 3, 4, 5]. Many of these complications stem from the indiscriminate thermal effects on nontarget tissue that is close to the targeted myocardium. Pulsed field ablation (PFA) on the other hand is a non-thermal ablative modality in which ultra-rapid high voltage impulses result in preferential myocardial tissue ablation [6, 7, 8, 9, 10]. An important level of tissue selectivity with PFA has resulted in reduced collateral damage compared to thermal energy, making PFA uniquely attractive [11, 12, 13, 14, 15, 16]. Numerous PFA technologies are currently under evaluation for AF ablation given their potential for improved procedural efficiency and safety (Table 1). PVI can be achieved via either a single-shot device or by focal catheter. Single-shot devices allow for a simpler procedural workflow, whereas focal, point-by-point PFA, allows for flexibility in lesion sets. We provide an overview of several single-shot and focal PFA catheters in this report.

**Table 1.** **Summary of PFA systems**.

	Medtronic	Medtronic/Affera		Boston/Farapulse	BiosenseWebster		Kardium	Adagio	Galaxy
Lesion set	Single-shot	Large focal	Single-shot	Single-shot	Single-shot	focal	Single-shot	Single-shot	any
Catheter	PulseSelect	Sphere-9	Sphere-360	Farawave	Varipulse	STSF	Globe	PFCA	any
3D map	NA	Affera	Affera	Rhythmia	CARTO	CARTO	Karidum	NA	any
Clinical trial	PULSED-AF	SPHERE-Per	FIH	ADVENT	inspIRE	smartIRE	PULSE-EU	FIH	ECLIPSE AF
PV durability w/OW	NA	PV: 97%, Patients: 90%	NA	PV: 96%, Patients: 84.1%	NA	NA	NA	NA	PV: ∼92%, Patients: ∼81%

FIH, First-In-Human; OW, optimized waveform; PFA, pulsed field ablation; PV, pulmonary vein; PFCA, pulsed field cryoablation; 3D, three dimensions; Pulsed-AF, Pulsed Field Ablation to Irreversibly Electroporate Tissue and Treat AF; SPHERE-Per, Treatment of Persistent Atrial Fibrillation With Sphere-9 Catheter and Affera Mapping and Ablation System; ADVENT, Randomized Controlled Trial for Pulsed Field Ablation versus Standard of Care Ablation for Paroxysmal Atrial Fibrillation; inspIRE, Study for Treatment of Paroxysmal Atrial Fibrillation by Pulsed Field Ablation System With Irreversible Electroporation; smartIRE, A Study For Treatment Of Paroxysmal Atrial Fibrillation With The THERMOCOOL SMARTTOUCH SF Catheter and TRUPULSE Generator; PULSE-EU, Safety and Performance of a Pulsed Field Device for Global Mapping and Ablation of the Left Atrium for the Treatment of Atrial Fibrillation; ECLIPSE AF, Safety & Clinical Performance Study of Catheter Ablation With the Centauri System for Patients With Atrial Fibrillation; NA, not applicable.

## 2. Single-Shot PFA Catheter Tips

**Fig. 1.**
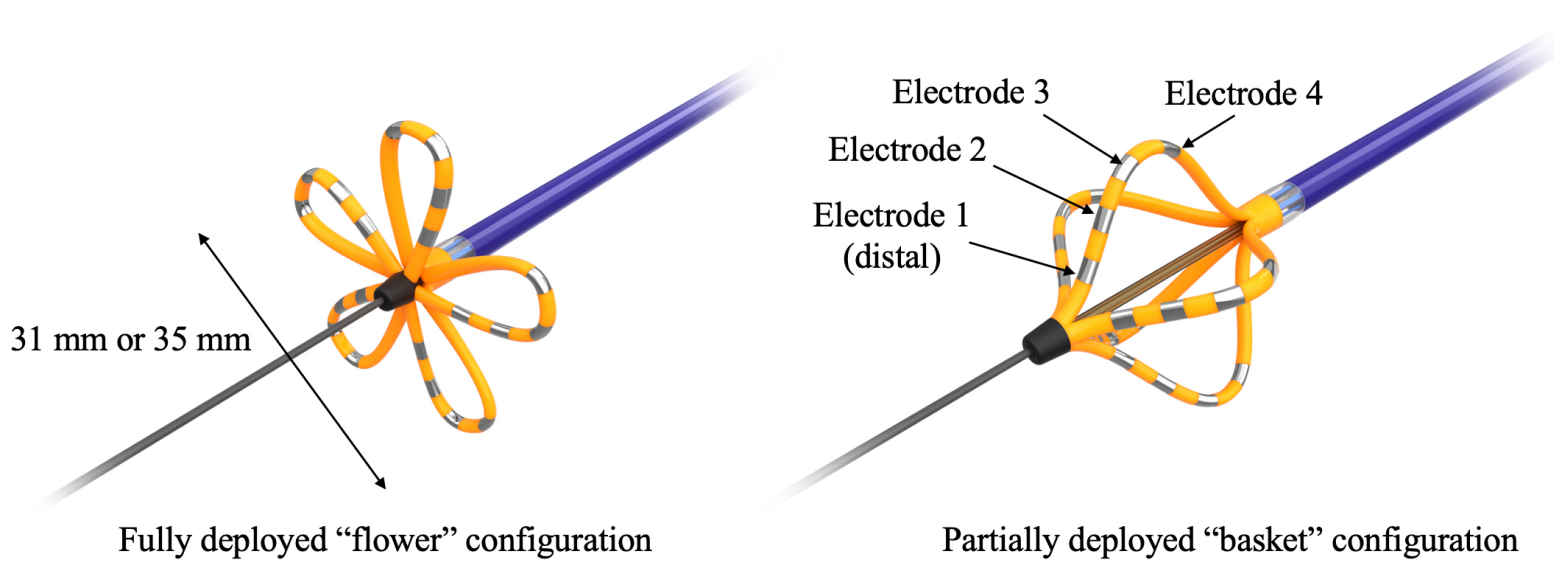
**Farawave catheter**. The 12.8 Fr over-the-wire ablation catheter is equipped with 5 splines containing 4 electrodes and can be deployed in either a basket or flower petal configurations enabling adaptable use within left atrial anatomy. All electrodes deliver ablative energy, with the third electrode on each spline individually wired for connection to a mapping or recording system.

The Farawave catheter (Farapulse-Boston Scientific Inc, Menlo Park, CA, USA) has 5 splines, each containing 4 electrodes, and can be deployed in either a basket or flower petal configurations, enabling adaptation to diverse left atrial and PV anatomies (Fig. 1) [17, 18]. The ablative energy is delivered from all electrodes with the third electrode on each spline separately wired to allow for mapping. The catheter was initially investigated in 2 clinical trials (*IMPULSE *and* PEFCAT*) for its safety and effectiveness in patients with paroxysmal AF using a custom generator that delivered either biphasic or monophasic waveforms in a bipolar fashion (Farastar, Farapulse-Boston Scientific Inc). The ablation protocol underwent consecutive evolutionary modifications: from monophasic to biphasic pulses, and then stepwise optimization of the biphasic waveform, pulse sequence and deployment strategy. Recent updated 1-year outcomes from a combination of three trials: *IMPULSE* (A Safety and Feasibility Study of the IOWA Approach Endocardial Ablation System to Treat Atrial Fibrillation), *PEFCAT* (A Safety and Feasibility Study of the FARAPULSE Endocardial Ablation System to Treat Paroxysmal Atrial Fibrillation) and *PEFCAT II * (Expanded Safety and Feasibility Study of the FARAPULSE Endocardial Multi Ablation System to Treat Paroxysmal Atrial Fibrillation ) demonstrated that acute PVI was achieved in 100% of PVs with PFA alone in 121 patients [19]. Durable PVI was achieved at 3 months follow-up in 96.0% of PVs (84.1% of patients) that were treated with the latest optimized biphasic waveform. Adjunctive ablation beyond PVI using this catheter has also been performed. A single-arm study of 25 patients with persistent AF (*PersAFOne: Pulsed Fields for Persistent Atrial Fibrillation*) was performed to evaluate PFA using the above system for both PVI and left atrial posterior wall isolation. In this report, acute PVI (96 of 96 PVs) and left atrial posterior wall (LAPW) ablation (24 of 24 patients) was acutely successful in all cases [20].

Recently,* MANIFEST-PF* (Multi-national survey on the methods, efficacy, and safety on the post-approval clinical use of pulsed field ablation) surveyed the performance of this system in routine clinical practice in Europe [21]. This retrospective survey included all 24 clinical centers using the catheter after its regulatory approval, including all patients treated at those centers since approval. The full cohort included 1758 patients with a mean age of 61.6 years, 57.5% were paroxysmal AF, and 34.2% were female. Acute PVI was successful in 99.9% with a mean procedure time of 65 min. There were no cases of esophageal complications, symptomatic PV stenosis, or phrenic nerve paralysis. Major complications occurred at a rate of 1.6%, with pericardial tamponade at 0.97% and stroke at 0.4%; one stroke resulted in fatality at 0.06%. Minor complications, totaling 3.9%, predominantly comprised vascular issues at 3.3%, alongside transient phrenic nerve paresis at 0.46% and transient ischemic attack at 0.11%. Rare complications, each occurring at a rate of 0.06%, included coronary artery spasm, hemoptysis, and persistent dry cough lasting for 6 weeks (n = 1 each). One-year follow-up data of the study demonstrated 81.6% and 71.5% of atrial arrhythmia freedom rates in paroxysmal and persistent AF patients, respectively [22].

There are currently two ongoing randomized control trials (*ADVENT* (Randomized Controlled Trial for Pulsed Field Ablation versus Standard of Care Ablation for Paroxysmal Atrial Fibrillation) and *BEAT-AF* (Ground-Breaking Electroporation-based intervention for Atrial Fibrillation treatment)) in US and Europe respectively that will further assess the safety and efficacy of this system [23]. In these studies, utilizing an optimized pulsed field (PF) waveform (1.8–2.0 kV), the process involves 2 applications to each PV in a partially open “basket” configuration, followed by rotation and an additional 2 applications. Subsequently, a second set of 2 applications in a fully deployed “flower” configuration (again with rotation between each pair of lesions) is conducted, resulting in a total of 8 PFA applications per PV. Most recently, the results from the *ADVENT* trial were reported. In this noninferiority trial, drug-refractory paroxysmal AF patients were randomly assigned to undergo PFA (n = 305) or thermal ablation (n = 302). There was no significant difference between PFA vs thermal ablation groups with regards to primary composite outcomes including an initial procedure failure (0.8% vs 0.8%), atrial tachyarrhythmia recurrence after a blanking period (17.2% vs 16.4%), antiarrhythmic drug use (8.1% vs 9.2%), cardioversion (0.5% vs 0.2%), or repeat ablation (0.5% vs 0.5%) and with regards to device- and procedure-related serious adverse events (2.1% vs 1.5%).

**Fig. 2.**
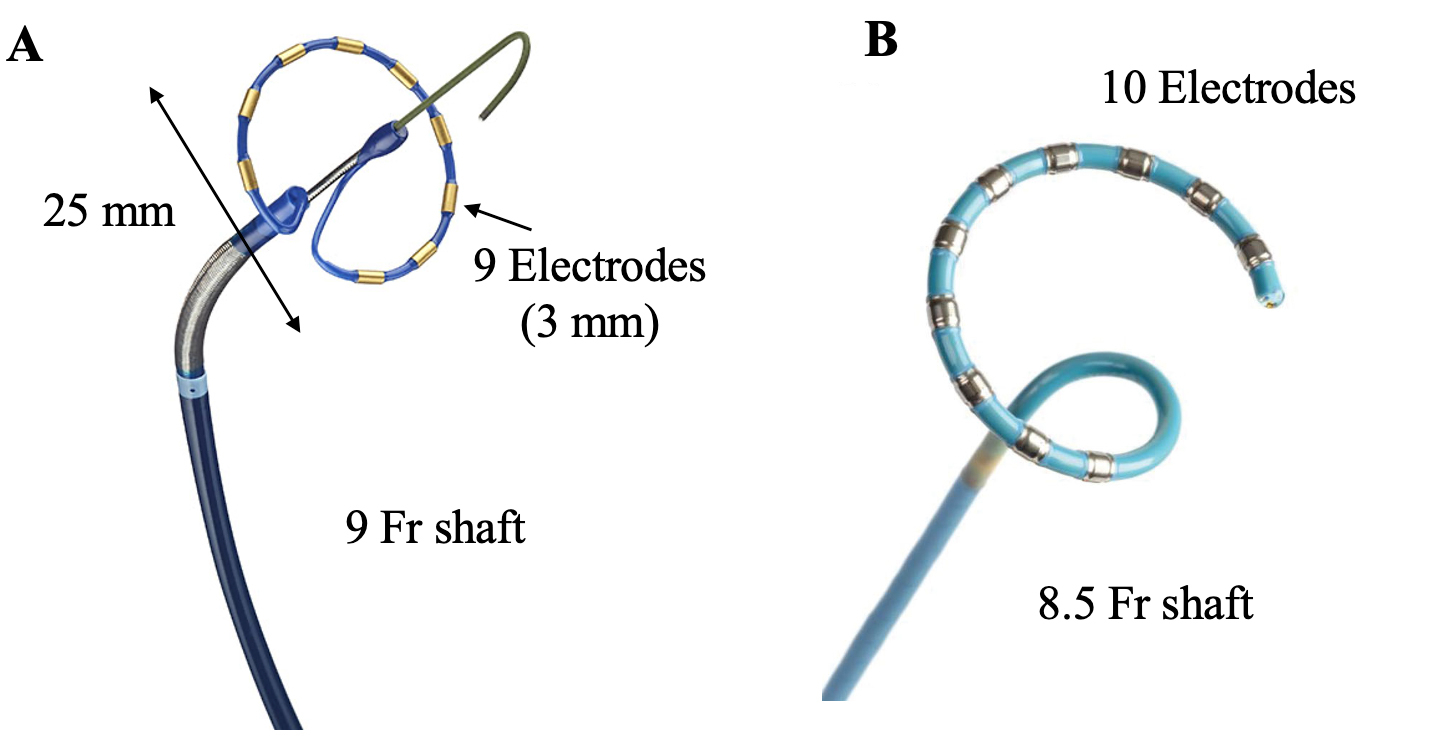
**PulseSelect and Varipulse catheters**. (A) PulseSelect catheter is a circular multielectrode array catheter with a 25 mm, 20° forward tilted array that mounts 9 gold electrodes. Each electrode is 3 mm in length and capable of recording as well as pacing. (B) Varipulse is an 8.5 Fr bidirectional, irrigated, variable-loop circular catheter that is capable of ablation as well as mapping with electroanatomical mapping system. The tip mounts with 10 platinum/iridium electrodes that are used for visualization, stimulation, recording, and bipolar PFA.

Several other single-shot PFA technologies are in development and evaluation. A circular multielectrode array catheter (PulseSelect; Medtronic, Inc, Fig. 2A) was evaluated in *PULSED AF* (*Pulsed Field Ablation to Irreversibly Electroporate Tissue and Treat AF*): a nonrandomized, prospective, multicenter, clinical trial [24]. A biphasic, bipolar waveform (500–1500 V) was delivered through a 25 mm, forward tilted array with 9 gold electrodes (9 Fr shaft and 25 mm in diameter). Each electrode is 3 mm in length and is capable of recording as well as pacing. Investigators initially described that all 152 PVs achieved acute electrical isolation in the 38 patients with a skin-to-skin procedure time of 160 ± 91 minutes. Left atrium (LA) dwell time averaged 82 ± 35 minutes, and fluoroscopy time averaged 28 ± 9 minutes. An average of 8.2 ± 4.2 energy applications were delivered around each of the PVs. No serious adverse events related to the PFA system was observed in any patients out to 30 days. More recently, updated results with a larger number of patients (paroxysmal AF; n = 150 and persistent AF; n = 150) were reported [25]. Acute PVI was achieved in all 300 patients. Each application contained 4 biphasic, bipolar pulse trains, lasting 100 to 200 ms at 1400–1500 V. Freedom from any atrial arrhythmia at 1 year follow-up was achieved in 69.5% for paroxysmal AF and 62.3% in patients with persistent AF. In both the paroxysmal and persistent AF cohorts, the primary safety endpoint was observed in 1 patient, accounting for 0.7% in each cohort (1 cerebrovascular accident and 1 pericardial effusion requiring drainage). Out of 45 patients who received cerebral magnetic resonance imaging (MRI) scans before and after ablation, 4 patients (8.9%) exhibited new silent cerebral lesions post-procedure, with a negligible change in Mini-Mental State Examination score. Additionally, among 63 patients who underwent imaging at baseline and 3-month follow-up, no significant pulmonary vein stenosis was observed.

Another PF technology that is currently being evaluated is an 8.5 Fr bidirectional, irrigated, variable-loop circular catheter (Biosense Webster, Diamond Bar, CA, USA, Fig. 2B) that is capable of ablation as well as mapping with electroanatomical mapping system (CARTO3 System; Biosense Webster) [26]. It has an adjustable circular tip with 10 platinum/iridium electrodes that are used for visualization, stimulation, recording, and bipolar PFA. A prospective, multicenter, single-arm clinical trial (*inspIRE*, *Study for Treatment of Paroxysmal Atrial Fibrillation by Pulsed Field Ablation System with Irreversible Electroporation (IRE)*) evaluated the above catheter as well as its compatible multi-channel generator (PFA Generator; Biosense Webster, Inc., Irvine, CA, USA). PFA was applied in a bipolar configuration (1800 V). Each PF application consists of sequences of biphasic pulses lasting microseconds, interspersed between them, summing up to a total application duration of ~250 ms. This study evaluated initial safety, including esophageal lesions, silent cerebral lesions, and PV stenosis in the ‘Wave I’ cohort (n = 40). The findings revealed no esophageal thermal lesions or PV stenosis. Among the 39 subjects who underwent cerebral MRI, silent cerebral lesions were identified in 4 of the initial 6 subjects. Subsequently, workflow enhancements, such as incorporating a 10-second pause between PFA applications, were implemented. This adjustment led to the detection of silent cerebral lesions in 4 of 33 subjects. In the following ‘Wave II’ phase (n = 186), no primary adverse events were reported. Achieving 100% entrance block, PVI without acute reconnection was successful in 97.1% of the targeted veins. One year freedom of any atrial arrhythmia was estimated in 70.9% of the patients.

**Fig. 3.**
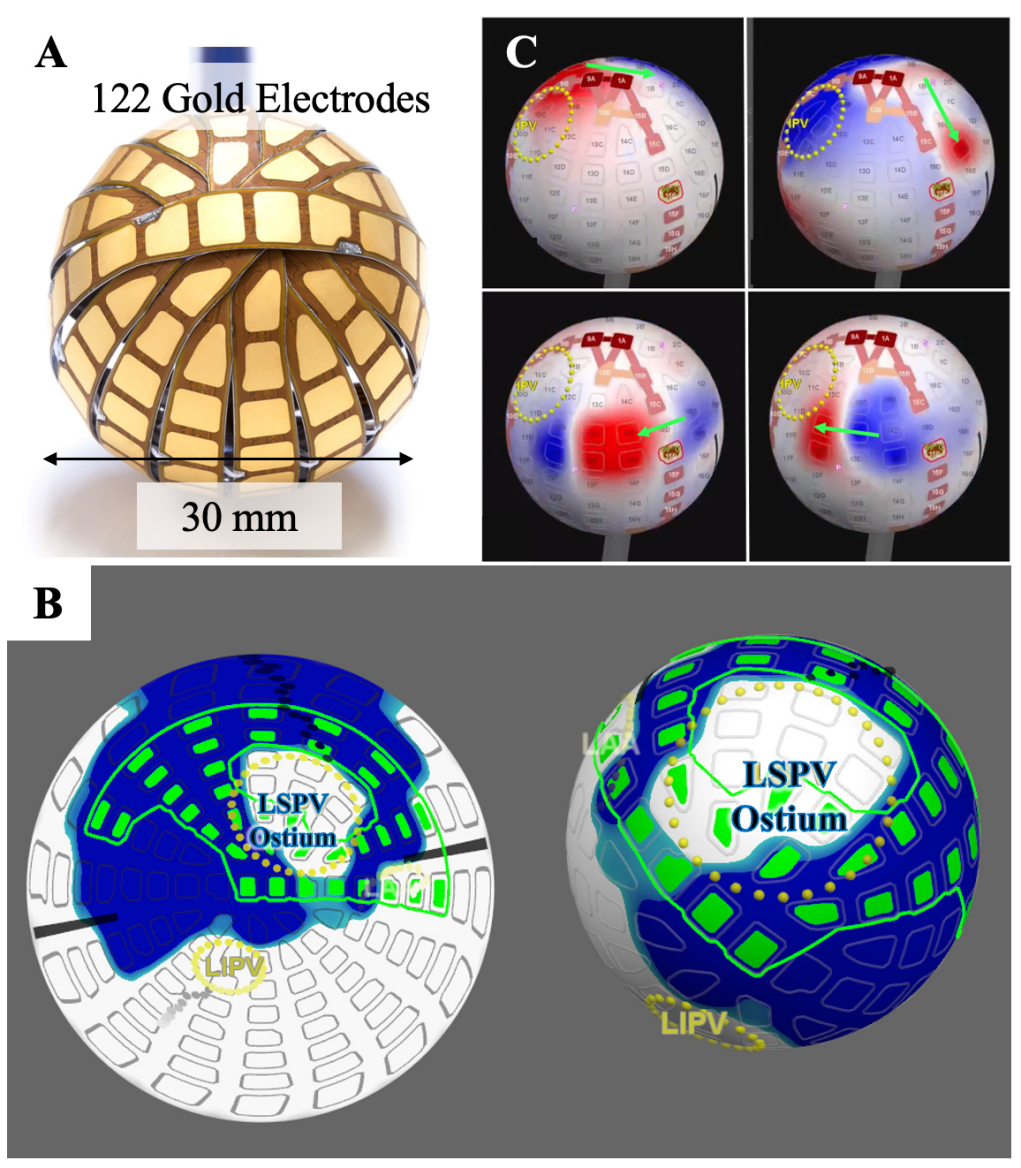
**Globe catheter**. (A) Globe catheter is fitted with a distal 30 mm multielectrode array, comprising 16 ribs equipped with 122 gold-plated electrodes. Each electrode serves multiple functions, allowing PFA/RFA, tissue contact measurement, temperature monitoring, current assessment, intracardiac electrogram recording, and application of stimulation pulses. (B) CONTACT Map is an updated representation of contact between the array’s electrodes and atrial tissue. Blue indicates good contact, while white denotes no contact. The 2D map is an inside-out view, while the 3D map is an outside-in view. Green electrodes are selected for ablation. (C) Wave Map (with explanatory arrows) showing gap identification. The lesion markers are RFA-based, and the gap was created intentionally. 2D, two dimensions; 3D, three dimensions; LIPV, left inferior pulmonary vein; LSPV, left superior pulmonary vein; PFA, pulsed field ablation; RFA, radiofrequency ablation; IPV, inferior pulmonary vein.

Another combined mapping and PFA catheter that is presently undergoing evaluation is a multielectrode spherical array (Globe, Kardium Inc, Burnaby, BC, Canada, Fig. 3A) [27]. The catheter is equipped with a distal 30mm multielectrode array that comprises 16 ribs upon which 122 gold-plated electrodes are positioned. Each electrode has multiple functions, enabling both ablation and the measurement of tissue contact, temperature, current, and intracardiac electrograms, and application of stimulation pulses. The Globe controller includes PF and radiofrequency (RF) generators, electronics for sensor data processing, and a stimulator for diagnostic purposes. The Globe console displays three dimensions (3D) electroanatomical maps and electrogram recordings from each electrode and enables control over the delivery of ablative energy to specific targets. The array’s electrodes are situated on flexible electronic circuits adhered to thin, rounded stainless steel ribs, averaging eight electrodes per rib. Temperature measurement at each electrode relies on a sensor positioned 0.025 mm directly behind it. The spacing between ribs permits blood flow through the device, even when positioned directly in front of a PV.

The data collected from the 122 electrodes generates various maps. The FLOW Map aids in resolving anatomical features such as pulmonary veins and determining electrode contact for ablation. It is a constantly updated depiction of contact between the array’s electrodes and the atrial tissue - blue signifies good contact, while white indicates no contact (Fig. 3B). Contact between the electrodes and the atrial wall is assessed through measurements of convective cooling by blood. A current is passed through the electrode temperature sensor, and the rate of cooling, as detected by this sensor, indicates if the electrode is in contact with tissue or cooled by flowing blood. The system is calibrated to measure different contact levels ranging from no contact to full electrode contact. The Voltage Map is a continually updated visual display representing the electrogram amplitude for each electrode. The WAVE Map demonstrates the propagation of the activation wavefront through the atrium, continuously updating to present the latest activation (Fig. 3C). It is created automatically by exhibiting the voltage measured at each electrode - blue indicates positive voltage, while red shows negative voltage. These visual presentations of electrograms allow continuous monitoring of activation propagation, even during ablation. For instance, this real-time visualization aids in observing PVI in real time during or post-ablation and is beneficial for identifying gaps in lesion lines.

The system can deliver either PFA or temperature-controlled RF. The 16 Fr catheter is inserted into the left atrium (LA) via a custom deflectable sheath. Once inside, coiled ribs are fanned open, forming a spherical array with a diameter of 30 mm. The initial findings from the first-in-human trial (*PULSE-EU*) demonstrated that patients who received an optimized PFA dose achieved 100% acute PVI and durable isolation in all cases at 3-month remapping. These results were achieved without instances of stroke/transient ischemic attack, pericardial effusion, phrenic nerve injury, or complications related to the esophagus [28]. The study expands on the successful Globe System *PULSE-EU* study of 69 patients and a larger study is being conducted in the USA (*PULSAR*).

**Fig. 4.**
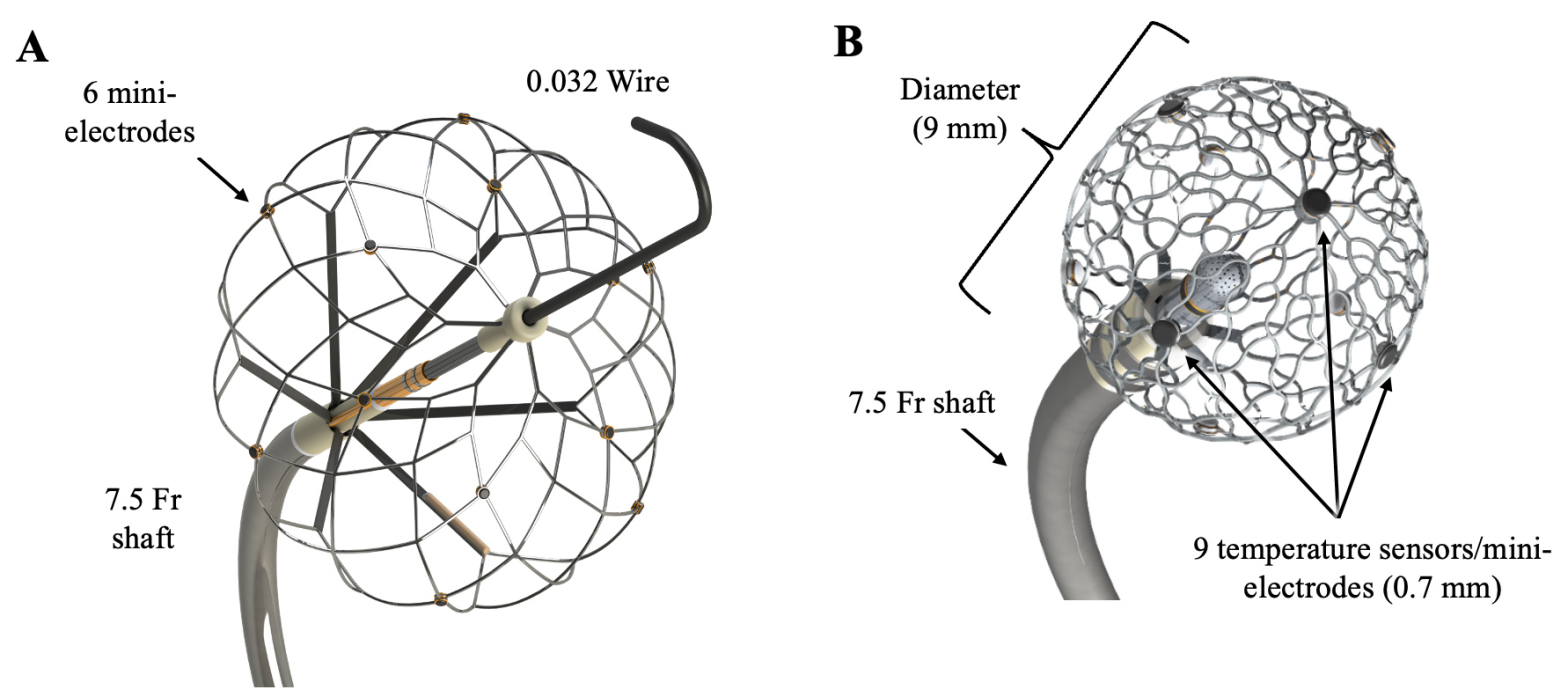
**Sphere-360 and Sphere-9 catheters**. (A) Sphere-360 has a large expandable (~34 mm), nitinol-based lattice framework tip that can be delivered through a standard 8.5 Fr steerable sheath over a 0.032 J-tipped wire in a collapsed form. The tip consists of six sections that can be independently and sequentially energized for ablation. (B) Sphere-9 is a 7.5 Fr catheter with a compressible spherical shaped 9-mm diameter nitinol lattice tip with 9 mini-electrodes (0.7 mm diameter each) and surface thermocouples were introduced. These two catheters are linked to an electroanatomical mapping system using a magnetic sensor.

Several other single-shot PFA devices are currently being investigated in early studies and we briefly describe a few such catheters for which preclinical data have been reported recently. Sphere-360 is a single shot design, that has a large expandable, nitinol-based lattice framework tip that can expand up to 34 mm in diameter (Affera-Medtronic Inc, Watertown, MA, USA, Fig. 4A). The expandable tip comprises six sections that can be independently and sequentially energized for ablation [29, 30]. The catheter can be inserted in a collapsed form using a standard 8.5 Fr steerable sheath over a 0.032 J-tipped wire. To achieve PVI, the wire tip is positioned in the distal PV, and the lattice tip is then expanded using the actuator in the catheter’s handle to optimize antral and circumferential contact. The catheter can be visualized in its compatible electroanatomical mapping system (Prism-1, Affera-Medtronic Inc., Newton, MA, USA) that has been previously described with the Sphere-9 catheter (Affera-Medtronic Inc, Watertown, MA, USA). Six distally placed microelectrodes are capable of recording bipolar or unipolar electrograms and PFA is delivered via the identical generator that powers the Sphere-9 catheter (HexaPULSE, Affera-Medtronic, Inc.). We and the other investigators reported 100% acute thoracic vein isolation (superior vena cava (SVC), left superior pulmonary vein (LSPV), and right superior pulmonary vein (RSPV)) with excellent durability in the chronic phase using the swine model [29].

Pulsed field cryoablation (PFCA) is a unique combination of ultra-low temperature cryoablation followed immediately by PFA, using the same catheter. Verma *et al*. [31] demonstrated optimized PFCA created transmural cavotricuspid isthmus lesions with evidence indicating tissue selectivity. This technique utilized an 8.5 Fr, non-deflectable catheter equipped with 20 electrodes and a central lumen. The catheter design allows the insertion of preconfigured, nonmalleable, nitinol stylets to give the catheter a specific shape (Adagio Medical) [31]. Ablations were performed using a custom PFA generator, ultra-low temperature cryoablation console, and an ablation catheter featuring insertable stylets. PFCA process involved precooling the tissue for 30 seconds followed by a concurrent biphasic, bipolar PFA train. The investigators also described that PFCA less produced heat formation and electrolysis as compared to PFA, suggesting that PFCA could allow for larger voltage deliveries while upholding the safety profile of PFA. Additional unique aspect of PFCA is its potential to reduce the risk of coronary spasm. Although PFA is attractive because of its tissue selectivity, the risk of coronary spasm needs to be carefully evaluated and monitored. The occurrence of coronary spasm was reported to occur after endocardial delivery using a contemporary biphasic PFA waveform both in a patient and in preclinical reports with histological changes of minimal intimal hyperplasia and medial fibrosis but with minimal to no loss of the lumen [32, 33, 34]. The mechanism of spasm remains unknown; one possibility could be the stimulation of smooth muscle cells from the excitatory nature of PF and/or activation of autonomic input, however coronary spasm is most likely a cross-effect of all PF modalities if the electrical field is high enough. A combination of PFA and cryoablation (PFCA) is one of the concepts to circumvent this issue. The increased impedance of cooled and frozen tissue might concentrate the electrical field in the low-temperature areas, potentially enhancing the selectivity of the PFA [35].

**Fig. 5.**
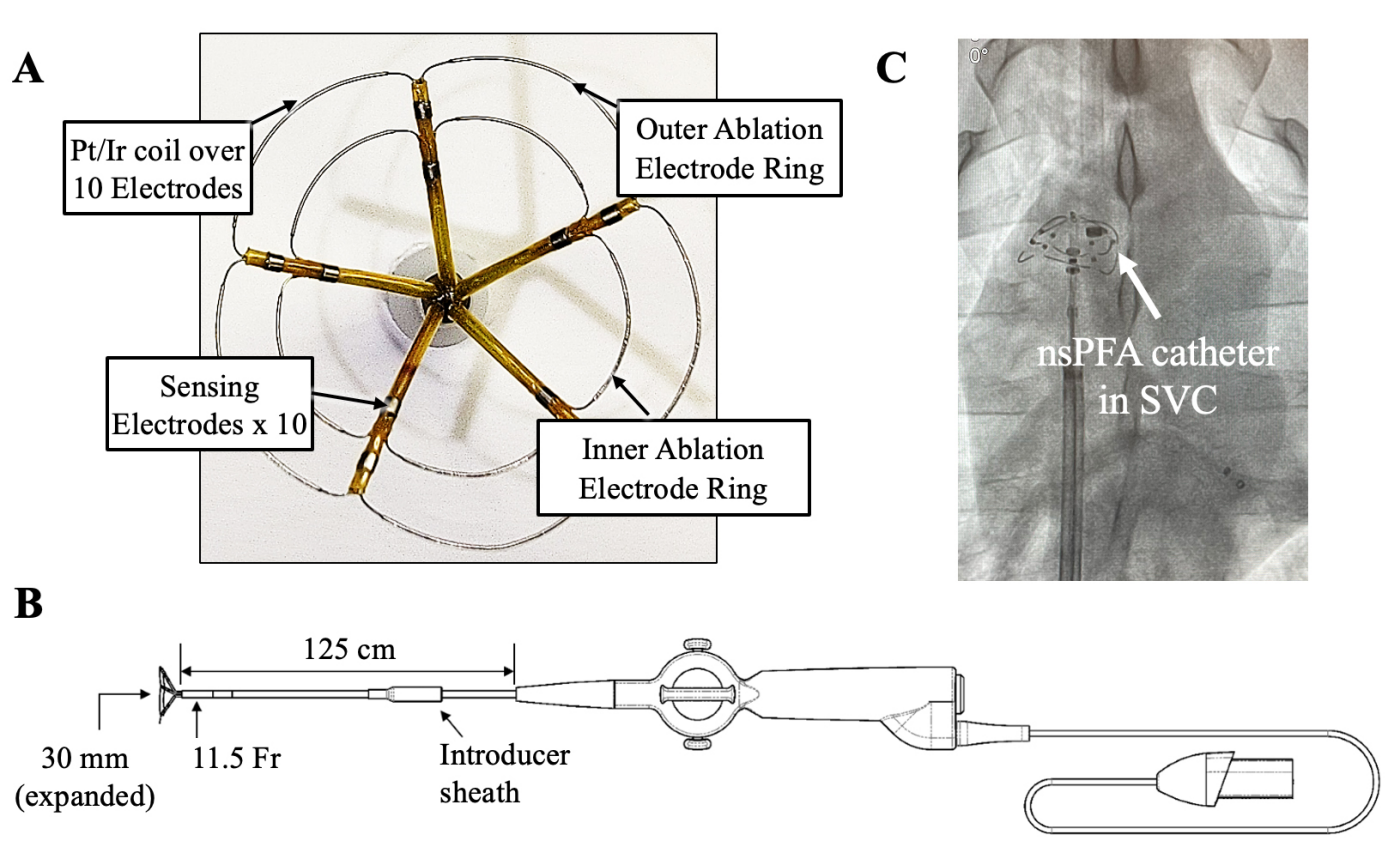
**CellFX catheter**. (A) CellFX catheter contains a 360-degree electrode array designed to allow for circumferential ablation in a single-shot manner. The array has a maximum diameter of 30 mm when fully expanded and contains two ablation electrode rings that deliver bipolar nsPFA energy directly to tissue along with 12 sensing electrodes for mapping. (B) The catheter consists of a 125 cm-long, 11.5 F shaft and a sensing cable that can connect to a compatible, commercially available mapping system. (C) Fluoroscopic image with CellFX catheter within SVC. nsPFA, nanosecond pulsed field ablation; Pt/Ir, Platinum Iridium; SVC, superior vena cava.

Another unique single-shot PFA device (CellFX, Pulse Biosciences, Inc, Fig. 5) that utilizes nanosecond PFA (nsPFA) is also undergoing preclinical evaluation [36]. In contrast to microsecond PFA, nsPFA utilizes pulse durations ~1000 times shorter and it allows pronounced permeabilizing effects on the organelle membranes. nsPFA may also improve ablation efficacy as well as reduce collateral muscle and nerve stimulation, which are important from a clinical workflow perspective. The catheter contains a 360-degree electrode array designed to allow for circumferential ablation in a single-shot manner. The electrode array has a maximum diameter of 30 mm when fully expanded and contains two ablation electrode rings that deliver bipolar nsPFA energy directly to tissue along with 12 sensing electrodes for mapping. The catheter consists of a 125 cm-long, 11.5 F shaft and a sensing cable that can connect to a compatible, commercially available mapping system. The integration of the sensing electrodes into the nsPFA catheter allows for a quick transition between mapping and ablation, without catheter withdrawal or repeat system setup. We evaluated its safety and effectiveness with a swine model. Ten swine underwent transfemoral venous access under general anesthesia. The nsPFA catheter was integrated with a custom electroanatomical mapping system (iMap; CardioNXT Inc, Boulder, CO, USA), and nsPFA lesions were delivered in both atria [37]. The catheter was well visualized within the mapping system and conformed well to venous ostia. All targeted atrial sites received successful nsPFA lesions: 10 in the SVC, 8 in the RSPV, and 4 in discrete atrial applications. Mild phrenic and muscular stimulation were observed. In one swine, 2 applications were necessary to improve RSPV antral coverage, and anatomical constraints prevented circumferential antral coverage in three other swine. Minimal PFA-related microbubbles were detected using ICE imaging. There were no instances of phrenic palsy, thrombus formation or ST segment elevation noted acutely or in follow-up. Necropsy findings indicated comprehensive, circumferential lesions in all 10 SVC (100%) and 5 of 7 RSPV (71%) targets. These lesions exhibited a distinctive halo-shaped appearance with a wide, continuous band encompassing a central dark zone. Transmural necrosis was seen in 21 of 22 (95.5%) lesions. The mean depth and width of SVC and RSPV lesions were 1.9 ± 0.4 mm, 14.1 ± 3.1 mm and 4.6 ± 2.6 mm, 15.5 ± 5.7 mm, respectively. All four discrete lesions were identified, reaching depths of up to 9.0 mm. The central dark core corresponded to a central hemorrhagic zone, indicating no evidence of thermal damage. Viable nerves and vessels were identified surrounding the nsPFA lesions.

## 3. Focal Point-by-Point PFA Catheter Tips

Although single-shot designs allow simpler work-flow approaches to achieving PVI, focal catheters capable of delivering PFA may be advantageous given their flexibility in delivering diverse lesion sets. In addition, focal catheters can be used for several non-PV ablation procedures in the atrium such as cavotricuspid isthmus, mitral isthmus, posterior wall isolation, and non-PV foci ablation. One such focal catheter- has a 7.5 F shaft and a compressible spherical shaped 9-mm diameter nitinol lattice tip with 9 mini-electrodes (0.7 mm diameter each) and surface thermocouples (Sphere-9; Affera-Medtronic, Inc, Fig. 4B). It has a central noncontact indifferent electrode within the lattice and 2 ring electrodes on the distal shaft. This bidirectional deflectable catheter can deliver either PF or RF using a custom generator (HexaGen and HexaPulse, Affera-Medtronic, Inc) and is linked to an electroanatomical mapping system (Hexamap, Affera-Medtronic Inc). Voltage and activation maps can be created simultaneously for anatomy acquisition. Each mini-electrode integrates a temperature sensor to facilitate temperature-controlled saline-irrigated RFA throughout the complete conductive lattice tip (the standard setting is 5 to 7 seconds with a target surface temperature of 73 to 75 °C). PFA is delivered in a monopolar fashion from the entire lattice tip (to a grounding electrode) using a biphasic waveform that consists of a train of microsecond-scale pulses delivered over 3 to 5 seconds, with total current delivery between 24 and 32 amperes. The dual-generator design (RF or PF) allows for effortless switching between RFA and PFA with a simple step on a foot pedal.

With the RF system, this focal catheter can create significantly larger lesions compared with a standard irrigation RF system. Because of the larger surface area of the spherical catheter (approximately 275 ${\mathrm{mm}}^{2}$) compared to a conventional irrigate RF catheter (approximately 28 ${\mathrm{mm}}^{2}$), resistive heating in the tissue is spread across a substantially larger and deeper area than occurs with a conventional RF ablation catheter [38]. The larger surface area also allows for a lower current density resulting a fewer occurrences of steam pop. Histological evaluation comparing PFA and RFA demonstrated that PFA lesions were replaced by homogenous fibrosis whereas coagulative necrosis was dominant in the RFA lesions [39]. With PF, repetition of applications creates larger lesions compared to a single application in the preclinical model (up to 9 mm) with an ability to penetrate epicardial fat and endocardial scarring up to 4 mm depth [40, 41].

A multi-center, single-arm, first-in-human trial was conducted to assess the safety and feasibility of this focal RF and PF system [42]. Toggling between energy sources, point-by-point PV encirclement was performed using biphasic PFA posteriorly and either temperature-controlled irrigated RFA (RF/PF strategy) or PFA anteriorly (PF/PF strategy). A total of 178 patients were included (70 paroxysmal and 108 persistent AF patients) and the waveforms evolved over time: PULSE1 (n = 76), PULSE2 (n = 47), and the optimized PULSE3 (n = 55). All lesion sets (comprising 78 mitral, 121 cavotricuspid isthmus, and 130 left atrial roof lines) were acutely successful. Invasive remapping conducted on 122 patients indicated an improvement in PVI durability with waveform evolution: PULSE1: 51%; PULSE2: 87%; and PULSE3: 97%. The one-year freedom from atrial arrhythmia was estimated at 78.3% and 77.9% for paroxysmal and persistent AF, respectively, and notably, 84.8% for the subset of persistent AF patients treated with the PULSE3 waveform. There was only one instance of a primary adverse event—inflammatory pericardial effusion that did not necessitate intervention [43].

Another focal system is the CENTAURI System (Galvanize Therapeutics)—this integrates its PFA generator to commercially available ablation catheters and mapping systems available in electrophysiology labs [44]. The system comprises 3 main components: the CENTAURI generator, delivering biphasic, monopolar PFA at selectable energy settings (19, 22, and 25 A); the CENTAURI Connect device enabling compatible focal ablation catheters and mapping systems connectivity; and a Cardiac Monitor (Ivy Biomedical Systems) synchronizing PFA delivery to the R-wave. *ECLIPSE AF* study (Safety & Clinical Performance Study of Catheter Ablation With the Centauri System for Patients With Atrial Fibrillation) was a prospective, single-arm, multi-center study assessing safety and acute, and chronic PVI durability utilizing the CENTAURI System alongside contact force-sensing focal ablation catheters and associated mapping systems: TactiCath SE and Ensite, StablePoint, and Rhythmia, and ThermoCool ST and CARTO. In a cohort of 82 patients, acute PVI was achieved in all pulmonary veins (322/322) with first-pass isolation in 92.2% (297/322). Invasive high-density remapping at 90 days verified that optimized PFA demonstrated per-patient isolation rates of approximately 81% and per-PV isolation rates of about 92%. Four serious adverse events were reported (three vascular access complications and one lacunar stroke), along with two incidents of catheter perforation.

Finally, we report another focal PFA utilizes a commercially available ablation catheter and mapping system. The ongoing *SmartfIRE *study—a prospective, multi-center, single-arm study that will enroll approximately 135 patients in Europe, is evaluating the safety and effectiveness of the ablation system (TRUPULSE Generator and the ThermoCool ST Catheter; Biosense Webster, Inc., Irvine, CA, USA) when used for isolation of the atrial PV in treatment of patients with paroxysmal AF. The dual energy generator can deliver RF or PF energy through the study catheter. The catheter and the generator are integrated with electroanatomical mapping system (CARTO3, Biosense Webster, Inc., Irvine, CA, USA) that allows real time visualization of the catheter.

## 4. Conclusions

Several PFA catheter ablation systems for atrial fibrillation ablation are under various stages of preclinical and clinical investigation. Each device has a proprietary PF waveform and a unique catheter design that allow for diverse lesions sizes and approaches to ablation. Most PFA systems have favorable acute success rates and low incidence of complications with short procedure times. However, clinical PFA studies in which chronic remapping was conducted demonstrated PV durability was improved with evolutional modifications of PF waveform. This suggests that this step may be critical to PFA catheters reaching a clinically effective dose and ablation strategy and must be considered for all catheters. PFA whether achieved by single-shot or focal point-by-point ablation, is a promising modality for safe and efficient pulmonary vein isolation and much needs to be learned from the ongoing real-world experience. This is especially true as unexpected and rare adverse events may surface only with increasing use and the overall impact of PFA on AF control may take some time to be determined.
